# Amiodarone-induced organizing pneumonia mimicking COVID-19: a case report

**DOI:** 10.1186/s40001-021-00522-w

**Published:** 2021-06-27

**Authors:** Gaetano Zizzo, Stefano Caruso, Elisabetta Ricchiuti, Roberto Turato, Ilario Stefani, Antonino Mazzone

**Affiliations:** 1 Department of Internal Medicine, Legnano and Cuggiono Hospitals, ASST Ovest Milanese, Milan, Italy; 2 Unit of Endoscopy, Cuggiono Hospital, ASST Ovest Milanese, Milan, Italy; 3 Division of Cardiorespiratory Medicine, Cuggiono Hospital, ASST Ovest Milanese, Milan, Italy

**Keywords:** Amiodarone, Toxicity, COVID-19, Pneumonia, ILD, Diagnosis, Swab, BALF, Lymphocytes, Eosinophils

## Abstract

**Background:**

Differential diagnosis of interstitial lung diseases (ILDs) during the COVID-19 pandemic is difficult, due to similarities in clinical and radiological presentation between COVID-19 and other ILDs on the one hand, and frequent false-negative swab results on the other. We describe a rare form of interstitial and organizing pneumonia resembling COVID-19, emphasizing some key aspects to focus on to get the right diagnosis and treat the patient properly.

**Case presentation:**

A 76-year-old man presented with short breath and dry cough in the midst of the COVID-19 outbreak. He showed bilateral crackles and interstitial-alveolar opacities on X-ray, corresponding on computed tomography (CT) to extensive consolidations with air bronchograms, surrounded by ground glass opacities (GGO). Although his throat-and-nasopharyngeal swab tested negative, the picture was overall compatible with COVID-19. On the other hand, he showed subacute, rather than hyperacute, clinical onset; few and stable parenchymal consolidations, rather than patchy and rapidly evolving GGO; pleural and pericardial thickening, pleural effusion, and lymph node enlargement, usually absent in COVID-19; and peripheral eosinophilia, rather than lymphopenia, suggestive of hypersensitivity. In the past year, he had been taking amiodarone for a history of ventricular ectopic beats. CT scans, in fact, highlighted hyperattenuation areas suggestive of amiodarone pulmonary accumulation and toxicity. Bronchoalveolar lavage fluid (BALF) investigation confirmed the absence of coronavirus genome in the lower respiratory tract; conversely, high numbers of foamy macrophages, eosinophils, and cytotoxic T lymphocytes with low CD4/CD8 T-cell ratio were detected, confirming the hypothesis of amiodarone-induced cryptogenic organizing pneumonia. Timely discontinuation of amiodarone and initiation of steroid therapy led to resolution of respiratory symptoms, systemic inflammation, and radiographic opacities.

**Conclusions:**

A comprehensive analysis of medical and pharmacological history, clinical onset, radiologic details, and peripheral and BALF cellularity, is required for a correct differential diagnosis and management of ILDs in the COVID-19 era.

## Background

Differential diagnosis of interstitial lung diseases (ILDs) has become challenging during the Coronavirus Disease 2019 (COVID-19) pandemic, especially in those regions most affected by the virus [1]. Nasopharyngeal swab for severe acute respiratory syndrome coronavirus 2 (SARS-CoV-2) detection, using reverse-transcription polymerase chain reaction (RT-PCR), can give false-negative results in a non-negligible percentage of cases [2–4], whereas imaging, in particular chest computed tomography (CT), may be suggestive for COVID-19 pneumonia (i.e., highly sensitive), yet not conclusive (poorly specific) [5, 6].

We describe a case of ILD that came to our observation in the midst of the COVID-19 outbreak in Lombardy (Northern Italy), which wants to be emblematic in this regard. In this patient, in fact, only the subsequent clinical course and in-depth research of SARS-CoV-2 genome in bronchoalveolar lavage fluid (BALF) allowed us to rule out the hypothesis of COVID-19 pneumonia or other infectious diseases, while an accurate analysis of medical history, clinical onset, radiologic features and BALF cytology finally oriented towards a correct approach to the patient’s pathology.

## Case presentation

**Fig. 1:**
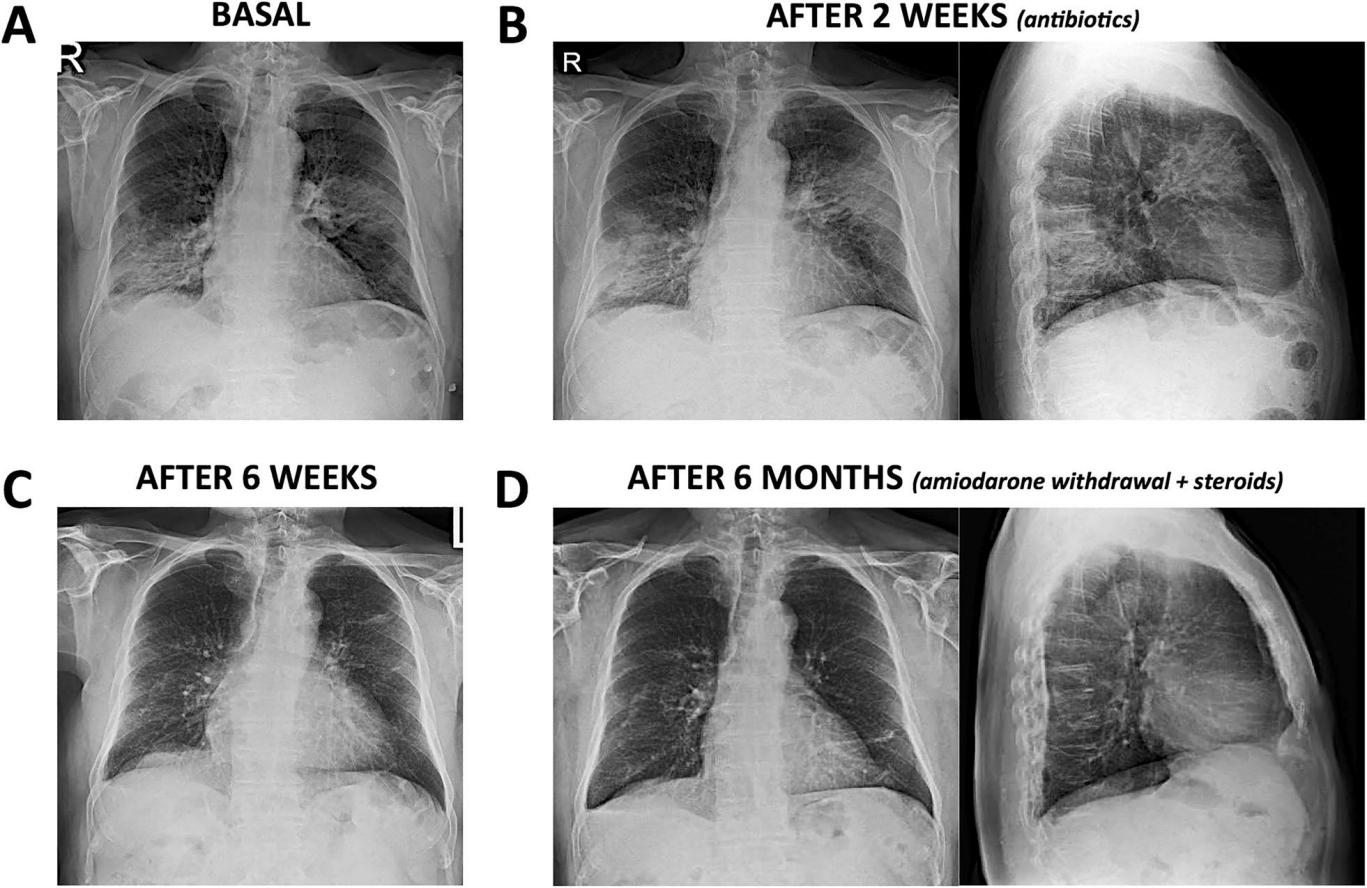
Chest X-rays. **A**, **B** Interstitial-alveolar opacities involving the posterior segment of the right lower lobe and the anterior segment of the left upper lobe; left hilar enlargement; and right basal pleural effusion. Baseline imaging was substantially unchanged after antibiotic therapy. **C**, **D** Progressive clearing and resolution of parenchymal opacities, with only right pleural thickening and costophrenic angle obliteration remaining after amiodarone discontinuation and steroid therapy

At the beginning of April 2020, a 76-year-old man presented to the emergency room of Magenta hospital (Milan) with worsening dyspnea and dry cough in the past 2 weeks. He was a former moderate smoker and a retired house painter. He denied fever, chest pain, lower limb edema or palpitations in the last period, although he had suffered from an unspecified arrhythmia a year earlier, for which he had to quit dancing. On auscultation he presented with bilateral, though localized, fine crackles. His chest X-ray showed extensive interstitial-alveolar opacities affecting the posterior segment of the right lower lobe and the anterior segment of the left upper lobe, morphologically compatible with COVID-19 pneumonia, and right basal pleural effusion (Fig. 1A, B). However, his combined throat-and-nasopharyngeal swab (RT-PCR) tested negative for SARS-CoV-2 infection. Blood tests at presentation demonstrated moderate leukocytosis (12,600/μL; reference range 4000–1000/μL), with neutrophilia (9000/μL; reference range 2000–7000/μL) and mild eosinophilia (700/μL; reference range 0–500/μL), normal lymphocyte count (1800/μL; reference range 1500–4000/μL), slight alteration of aspartate aminotransferase (61 U/L; normal values ≤ 45 U/L), and substantial C-reactive protein (CRP) elevation (11.4 mg/dL; normal values ≤ 0.5 mg/dL). Gas analysis showed modestly impaired blood oxygen level, with an arterial partial pressure of oxygen (PaO2) of 66.4 mmHg (reference range 80–100 mmHg) and an arterial oxygen saturation (SaO2) of 93.5% (reference range 95–100%), no signs of acute respiratory distress syndrome (ARDS) (ratio of PaO2 to the fraction of inspired oxygen or P/F ratio = 316.2 mmHg; reference range 400–500 mmHg; values indicative of acute lung injury and ARDS ≤ 300 mmHg), and values of pH, arterial partial pressure of carbon dioxide and lactates ranging within limits. He was transferred to the nearby Cuggiono hospital in low-flow nasal cannula oxygen therapy for further investigation and treatment.

**Fig. 2:**
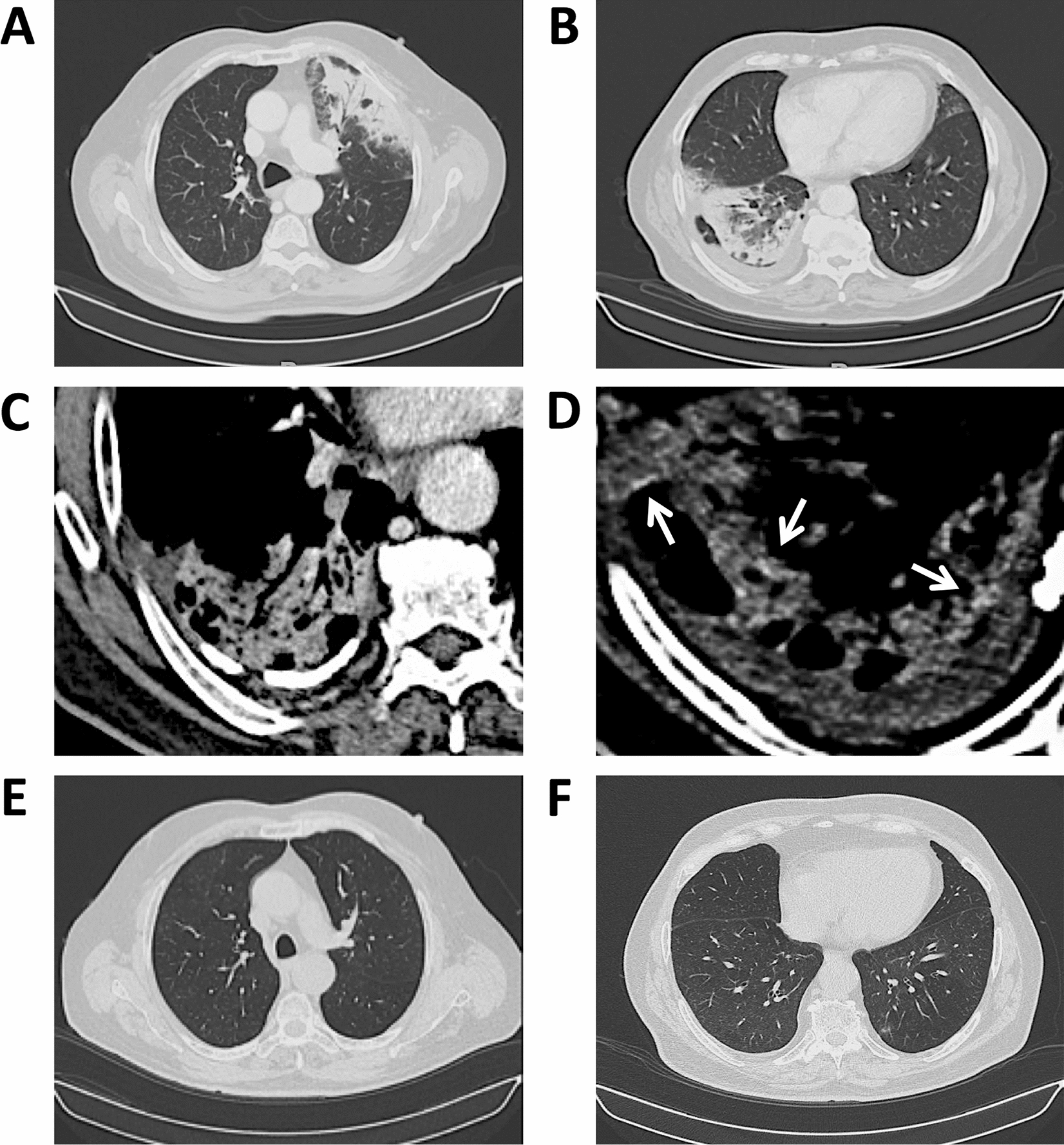
CT scans. **A** Left upper lobe consolidation with air bronchograms surrounded by ground glass opacities; left hilar lymphadenopathy. **B** Right lower lobe consolidation delimited by right oblique fissure with air bronchograms; right basal pleural effusion; left anterior pericardial thickening (*lung windows*). **C** Right posterior pleural thickening and calcification. **D** Areas of hyperattenuation (110–125 Hounsfield units) (*arrows*) possibly caused by iodine accumulation (*mediastinal windows*). **E**, **F** Complete resolution of parenchymal consolidations and pleural effusion following treatment with glucocorticoids and amiodarone wash-out

High-resolution CT (HRCT) scans showed bilateral and subpleural pulmonary consolidations with air bronchograms, surrounded by ground glass opacities (GGO) (Fig. 2A, B). A moderate hilar reactive lymphadenopathy, a partly calcific thickening of the right costal pleura with pleural effusion in the costovertebral groove, and a modest left anterior pericardial thickening were also observed (Fig. 2A–C).

Empirically, he was initially treated with a broad-spectrum antibiotic therapy (meropenem 1 g/8 h and azithromycin 500 mg/24 h) for 2 weeks, with poor clinical benefit on crackles and unchanged control chest radiography (Fig. 1B), although pulmonary gas exchange slightly improved (PaO2 69.9 mmHg, SaO2 94.7%) and he was weaned off oxygen. Control blood tests showed partially reduced, still persistently high, CRP levels (8.5–8.6 mg/dL), with further increasing eosinophil counts (up to 1200/μL, and 11.8% of total leukocytes). Serum galattomannan (Aspergillus) antigen and anti-neutrophil cytoplasmic antibodies (ANCA) tested negative, and he underwent bronchoalveolar lavage for further investigation. Cultures from BALF did not grow any bacteria, fungi or mycobacteria, and no cancer cells were found in BALF specimens. Notably, RT-PCR analysis on BALF samples and serological chemiluminescent immunoassay were confirmed negative for SARS-CoV-2 infection and SARS-CoV-2 antibodies. BALF showed markedly increased cellularity (1,159,000 cells/mL; reference range 80,000–200,000/mL) and was highly enriched in lymphocytes (24.7%; normal values ≤ 10%), with large predominance of CD8^+^ T cells (CD4/CD8 ratio = 0.2; reference range 1.2–1.8), and milder increase in neutrophils (3.2%; normal values < 3%) and eosinophils (3.9%; normal values a < 2%); moreover, approximately one third of total macrophages (65.7%; normal values ≥ 80%) displayed a foamy aspect (21.3%).

Given the presence of eosinophilia and the massive alveolar infiltration by cytotoxic T lymphocytes (CTL), we finally tested the hypothesis that such form of organizing pneumonia was due to some chronic drug exposure and toxicity. For years, the patient was taking dutasteride and tamsulosin for prostatic hyperplasia, and cyclic rifaximin therapy for diverticular disease. Remarkably, in the last year, he was also taking amiodarone for an arrhythmia that he could not better specify. His electrocardiogram reported normal sinus rhythm with QT interval falling within limits, blood concentration of N-terminal prohormone of brain natriuretic peptide was normal, and his echocardiogram did not show significant abnormalities. His family was then asked to bring previous cardiological documentation. We, therefore, ascertained that the patient had suffered from ventricular ectopic beats after exertion, with numerous extrasystoles being reported at Holter monitoring. Echocardiography had ruled out the presence of structural heart disease, and a stress test had shown no inducible ischemia. Assuming it was a cryptogenic organizing pneumonia (COP) secondary to amiodarone, after having consulted a cardiologist, amiodarone was withdrawn and replaced with low doses of bisoprolol. Moderate doses of prednisone (37.5 mg per day, corresponding to ≅ 0.5 mg/kg/day) were then started, to be tapered over at least 6 months. Abstention from excess physical activity and regular control of blood pressure and heart rate were adviced, with cardiological re-evaluation after few weeks. Bisoprolol was soon stopped for bradycardia, and a new Holter was planned after a congruous amiodarone washout period; thyroid-stimulating hormone, lactate dehydrogenase, creatine kinase and transaminases were normal.

An X-ray repeated 6 weeks later already showed complete resolution of the parenchymal opacity in the right lower field, with no pleural effusions, and a residual small area of ​​parenchymal thickening in the left upper field (Fig. 1C). After 6 months, all lung fields were clear, with only pleural thickening remaining on the right lung (Fig. 1D).

The patient reported symptom resolution with no side effects, subjective well-being, and normal pulse oxymetry (peripheral oxygen saturation = 98%). Blood tests showed normal leukocyte and eosinophil counts (9200–9300/μL and 200/μL, respectively), with normal CRP levels (0.1–0.3 mg/dL) and low erythrocyte sedimentation rates (4–5 mm/h; normal values ≤ 15 mm/h). A control HRCT scan performed at 8 months (while taking 6.25 mg of prednisone per day, < 0.1 mg/kg/day) confirmed stable remission of lung consolidations (Fig. 2E, F), thus allowing steroid discontinuation.

## Discussion and conclusions

Accuracy in the diagnosis of COVID-19 and in the differential diagnosis of ILDs during the COVID-19 outbreak is still an unmet need. RT-PCR assays have shown high rates of false-negative results when searching SARS-CoV-2 genome in the upper respiratory tract [2–4, 7]. Sensitivity of nasopharyngeal swabs has increased over the time of the pandemic, from 45–65% in the first months [2, 3] to approximately 75–85% later [8, 9]. Despite improved methodology, sensitivity of RT-PCR tests still remains substantially affected by timing. Nasopharyngeal swabs show the best sensitivity in the first week after symptom onset (80–85%), yet they show low sensitivity during disease incubation (up to 100% of false-negative results), and in the second and third week after symptom onset (from one third to two thirds of false-negative results, respectively) [4, 10].

Given its high sensitivity, CT is used as an important complement to the nasopharyngeal swab for diagnosing COVID-19; however, it also shows low specificity [5, 6]. Therefore, it is critically important to examine additional diagnostic clues. Here, we highlight the key contribution of different items, namely medical history, clinical presentation, radiologic details, and BALF data, to make a correct differential diagnosis of ILDs during the pandemic.

The patient we describe may have easily been misdiagnosed as having COVID-19 pneumonia with a false-negative swab. In fact, he presented in the midst of the COVID-19 outbreak with worsening dyspnea and dry cough, had variable increase in peripheral leukocytes, C-reactive protein and transaminases, and showed interstitial lung thickening with bilateral and subpleural GGO and consolidations.

On the other hand, he also presented with distinctive clinical, radiological and laboratory features pointing to alternative diagnostic hypotheses, especially in light of his recent exposure to amiodarone (Table 1). Clinically, the subacute presentation, in the absence of acute respiratory failure (P/F ratio > 300 mmHg), fever, fatigue or myalgia, contrasted with the acute or hyperacute onset characteristic of viral respiratory diseases such as COVID-19, and was consistent with the progressive tissue accumulation of amiodarone in the last year and correlation of amiodarone-induced pulmonary toxicity (APT) with its cumulative dose [11–14]. In fact, whereas ARDS is frequently observed in COVID-19, amiodarone-induced ARDS has been described only occasionally, and specifically in patients undergoing cardiothoracic surgery [12]. Furthermore, in our patient, amiodarone may have caused a latent cardiac toxicity, unmasked by his tendency to bradycardia while taking low doses of bisoprolol [12].

**Table 1:** Comparison between Amiodarone-induced and COVID-19 pneumonia

	Amiodarone-induced organizing Pneumonia	COVID-19 Pneumonia
Clinical onset	Subacute or chronic (drug accumulation over 6–12 months)	Acute or hyperacute (rapid evolution)
Short breath	Common	Common
Dry cough	Common	Common
Fever	Absent or mild	Moderate or high
Fatigue and Myalgia	Absent or mild	Severe and debilitating
Other symptoms	Bradycardia, thyroid dysfunction, blue-grey skin, etc	Anosmia, conjunctivitis, diarrhea, etc
Lactate dehydrogenase and transaminase elevation	Absent or mild	Moderate or severe
Hypoxia	Absent or mild (P/F ratio > 300 mmHg)	Moderate or severe (P/F ratio ≤ 200–300 mmHg)
ARDS	Rare (after cardiothoracic surgery)	Frequent
Bilateral pneumonia	Common (right and upper lobes)	Common (lower lobes)
Interstitial and alveolar opacities	Common	Common
Consolidations with air bronchograms	Predominant	Frequent
Ground glass opacities	Frequent	Predominant
Multifocal distribution	Fewer segments and lobes involved	Many segments (≅ 6) and lobes (≅ 3) involved (patchy distribution)
Hyperattenuation on CT	Frequent (iodine accumulation)	Absent
Pleural thickening (and effusion)	Frequent	Absent or rare
Pericardial thickening	Frequent	Absent or rare
Lymphoadenopathy	Frequent (moderate and reactive)	Absent or rare (lymph node atrophy)
Response to steroids	Good	Variable
Pulmonary thrombosis	Rare	Frequent
Peripheral leukocytosis	Frequent	Frequent
Peripheral neutrophilia	Frequent	Common
Peripheral eosinophilia	Common	Absent or rare
Peripheral lymphopenia	Absent	Common
Alveolar hypercellularity	Common	Common
Alveolar neutrophilia	Frequent	Common
Alveolar eosinophilia	Common	Absent or rare
Alveolar lymphocytosis	Common	Absent or variable
Alveolar CD8^+^ T cells	Increased	Decreased or variable
Alveolar foamy macrophages	Common	Absent or variable
RT-PCR on BALF samples	Negative	Positive for SARS-CoV-2 RNA

P/F ratio: arterial partial pressure of oxygen divided by the fraction of inspired oxygen; ARDS: acute respiratory distress syndrome; CT: computed tomography; RT-PCR: reverse transcriptase polymerase chain reaction; BALF: bronchoalveolar lavage fluid; SARS-CoV-2 RNA: severe acute respiratory syndrome coronavirus 2 ribonucleic acid

Radiologically, the patient basically showed two large pulmonary consolidations with air bronchograms, already present at clinical onset and stable over at least 2 weeks, thereby compatible with amiodarone-induced COP [15]. By contrast, COVID-19 pneumonia is generally characterized by multifocal patchy distribution involving many lobes and segments, rapidly confluent GGO, and more extensive GGO than consolidations [16, 17]. Remarkably, pleural and pericardial thickening, in some cases with pleural effusion as in our patient, are frequently observed after amiodarone exposure [12–15], but not in COVID-19 [16–18]. Similarly, lymph node enlargement, even moderate as seen in our patient, is not typical of COVID-19 [16–18], in which a diffuse lymphopenia is indeed associated with the atrophy of secondary lymphoid organs [19]. Additional radiological details pointing to the diagnosis of APT and COP, rather than COVID-19, were the preponderant involvement of the right lung and pleura, the presence of hyperattenuation areas (Fig. 2D), and the complete clearing of radiographic opacities upon treatment with moderate doses of steroids, as previously described [12, 14, 15, 20].

Blood tests were also suggestive in this regard, highlighting the presence of eosinophilia, rather than lymphopenia, a milder neutrophilia and a lower neutrophil-to-lymphocyte ratio as compared to COVID-19, and only a transient and non-specific rise in aspartate aminotransferase level [12, 15, 16, 19].

Our report strongly supports the use of bronchoscopy with bronchoalveolar lavage as a valuable tool for discriminating COVID-19 pneumonia from other ILDs. BALF analysis is desirable for an in-depth search of SARS-CoV-2 genome when the clinical and radiological picture is suggestive of COVID-19 pneumonia but the nasopharyngeal swab is negative, due to the higher sensitivity of RT-PCR testing in BALF specimens (up to 93–100%) [4, 7]. BALF investigation also allows to rule out other infections or malignancies. Most remarkably, BALF provides precious information about alveolar cellularity. In our patient, the presence of eosinophilia, in peripheral blood (i.e, eosinophils > 1000/μL) as at the alveolar level (ie, eosinophils > 2–3% of total leukocytes), was strongly suggestive of drug-induced pneumonia or hypersensitivity pneumonitis. As generally observed for APT [15], alveolar eosinophil count did not reach the diagnostic criteria for eosinophilic pneumonia (i.e., eosinophils > 25%), and other causes of pulmonary eosinophilia, such as asthma, fungal or mycobacterial infections, and ANCA-positive eosinophilic granulomatosis with polyangiitis were excluded. Furthermore, the conspicuous presence of foamy macrophages in the patient’s BALF importantly reflected the typical “amiodarone effect” observed on phospholipid metabolism, the lack of which made the hypothesis of APT very unlikely [12, 13, 15]. Conversely, the high contents of CD8 T cells in the patient’s alveoli was highly suggestive in this regard [13, 15]. In fact, a low CD4/CD8 T cell ratio in BALF or an overt lymphoid alveolitis (i.e., lymphocyte count > 15%) with preponderance of CTL, either isolated or associated with eosinophilic or neutrophilic alveolitis, have been described as key cytological characteristics of amiodarone-induced pneumonia [21], and are consistent with our experience. By contrast, in progressing COVID-19 pneumonia, alveolar CD8 T cells, although phenotypically activated in a proinflammatory manner, decrease in numbers, in parallel with general lymphopenia [22, 23].

Obviously, amiodarone-induced COP is a diagnosis of exclusion [13, 15]; on the other hand, a number of elements strongly suggest this hypothesis, including subacute presentation and temporal relationship to amiodarone administration, radiologic aspects, cytological findings, and response to therapies. While the partial reduction of CRP levels and the modest improvement in respiratory exchanges after antibiotic therapy, in the absence of microbiological findings, might have been ascribed to discrete anti-inflammatory effects of azithromycin (e.g., on mitogen-activated protein kinases [24]), only the timely withdrawal of the pneumotoxic agent (amiodarone) and addition of steroids in adequate starting doses (prednisone 0.5 mg/kg/day) [12, 14, 15] finally led to prompt and complete resolution of pulmonary consolidations, with normalization of oxygen exchange, and drastic reduction of eosinophilia and inflammatory indices. Furthermore, the fact that the slow tapering of steroids was able to protect from disease relapse is consistent with the long elimination half-life of amiodarone, which may actually persist in the body for up to 6–8 months [13].

In conclusion, the COVID-19 pandemic is severely challenging clinicians’ ability to differentially diagnose interstitial and organizing pneumonia. Our case report emphasizes the importance of carefully considering (a) medical history (including past and pharmacological history), (b) clinical presentation (e.g., subacute *vs.* acute or hyperacute onset), (c) radiological findings (e.g., predominance of parenchymal consolidations *vs.* patchy GGO, presence *vs.* absence of pleural or pericardial or lymph node thickening, specific details such as amiodarone-associated hyperattenuation), and (d) BALF study (for in-depth research of coronavirus, exclusion of other causes, and analysis of cellularity and CD4/CD8 T cell ratio at the alveolar level), to correctly guide the physician in the management of ILDs in the “COVID-19 era”.

## Data Availability

Clinical, laboratory and instrumental data pertaining to the patient are available from the corresponding author on reasonable request.

## Abbreviations

ANCA: Anti-neutrophil cytoplasmic antibodies
APT: Amiodarone-induced pulmonary toxicity
ARDS: Acute respiratory distress syndrome
BALF: Bronchoalveolar lavage fluid
CD: Cluster of differentiation
COP: Cryptogenic organizing pneumonia
COVID-19: Coronavirus Disease 2019
CRP: C-reactive protein
CT: Computed tomography
CTL: Cytotoxic T lymphocytes
GGO: Ground glass opacity
HRCT: High-resolution computed tomography
ILD: Interstitial lung disease
PaO2: Arterial partial pressure of oxygen
P/F ratio: Arterial partial pressure of oxygen divided by the fraction of inspired oxygen
RT-PCR: Reverse transcriptase polymerase chain reaction
SaO2: Arterial oxygen saturation
SARS-CoV-2: Severe acute respiratory syndrome coronavirus 2
